# Foundations for consensus on establishing a unified definition of death: results of the European Society for Organ Transplantation Bucharest International Consensus Conference

**DOI:** 10.3389/ti.2026.16498

**Published:** 2026-09-01

**Authors:** Francesco Procaccio, Alexandra Glazier, Beatriz Domínguez-Gil, Corinne Antoine, James Bernat, Helen Opdam, Mario Royo-Villanova, Gabriel C. Oniscu, Umberto Cillo, Dominique E. Martin

**Affiliations:** 1 Fondazione Trapianti, Milan, Italy; 2 New England Donor Services, Waltham, MA, United States; 3 Brown University, Providence, RI, United States; 4 Organizacion Nacional de Trasplantes, Madrid, Spain; 5 Agence de la Biomedecine, Saint-Denis, France; 6 Dartmouth College Geisel School of Medicine, Hanover, NH, United States; 7 Australian Organ and Tissue Authority, Canberra, ACT, Australia; 8 Department of Intensive Care, Austin Hospital, Heidelberg, VIC, Australia; 9 Virgen de la Arrixaca University Clinical Hospital, Murcia, Spain; 10 Division of Transplantation Surgery, CLINTEC, Karolinska Institutet, Stockholm, Sweden; 11 Unità di Chirurgia Epatobiliare e Trapianto Epatico, Università degli Studi di Padova, Padova, Italy; 12 School of Medicine, Deakin University. Geelong, VIC, Australia

**Keywords:** DCDD (donation after circulatory determination of death), determination of death, ethics, consensus conference, deceased donation

## Abstract

As part of the ESOT International Consensus on Controlled Donation After Circulatory Determination of Death (cDCDD) Project, a dedicated working group addressed considerations relating to the determination of death in the context of cDCDD. Delphi methodology was used to explore international perspectives on the definition of death, and clinical and ethical standards for the determination of death in the setting of cDCDD. The results indicated consensus that establishing a “single unified definition of death worldwide” and specifically the “unifying brain-based concept of death” would be beneficial. There was consensus on the proposed definition of this concept and several principles relating to the determination of death in the context of cDCDD, such as the “dead donor rule.” However, limited agreement was reached on the more practical aspects of death determination. The results nevertheless provide a foundation for current policy and practice and should guide further efforts to establish minimum clinical standards for the determination of death in the context of cDCDD.

## Introduction

Defining death and determining death have long been the subject of academic and clinical debate, given their profound relevance across medicine, ethics, law and society. Clearly establishing when death has occurred is essential not only to respect the dignity and wishes of the dying individual, but also to provide certainty for families, guide timely end-of-life and post-mortem decisions and maintain public trust in medical institutions. As technological innovations evolve to sustain vital functions artificially, identifying the boundary between life and death is dependent on increasingly specific clinical criteria, making a widely accepted operational definition of death more critical than ever [1, 2].

Defining death is central to the field of deceased organ donation and transplantation. Clarity in the determination of death in the context of deceased donation is important to ensure adherence to the dead donor rule (DDR) -- the ethical principle that the recovery of a donor’s organs must not be the cause of death and therefore prospective donors must be declared dead prior to the surgical recovery of vital organs. Additionally, the advent of new technologies and protocols for the recovery, preservation and repair of organs obtained from deceased donors expands opportunities for donation and transplantation, but also raises new questions under existing definitions of death with important implications for clinical practice [3]. In the context of controlled donation after circulatory determination of death (cDCDD), an accurate and timely determination of death is critical to maximize a successful donation process [1]. Global consensus on defining death and clinical standards for determining death are widely recognized as necessary to support the development and expansion of cDCDD programs in an ethically principled manner [4]. In designing the international DCD Consensus Project, the European Society for Organ Transplantation (ESOT) therefore established a working group on the topic of death, with a particular focus on questions regarding the unifying brain-based concept of death within the context of cDCDD [5]. This concept has been proposed as a possible foundational pathway for international consensus to harmonize policy, support essential principles such as the DDR, and facilitate current practice in deceased donation [6].

## Methods

The Delphi process applied in the overarching DCD Consensus project is outlined in more detail elsewhere [5]. A steering committee comprised of eight members with expertise in neurology, intensive care, law, and ethics developed a questionnaire addressing a range of topics identified as priorities via literature review and group discussions, and through consultation with members of the Adult and Paediatric Pathway subgroups [7, 8]. The committee identified expert panellists according to the criteria described in Martin et al. who were invited to participate in the Delphi process [5]. The two coordinators were excluded from the expert panel.

In each survey round panellists indicated their level of agreement with a series of statements using a Likert scale (1–9, strongly disagree–strongly agree); responses were analysed using descriptive statistics with “disagreement” assigned to ratings 1–3, “neither agree nor disagree” 4–6, and ‘agreement’ to ratings 7–9. Participants could alternatively indicate if a question was not relevant to their expertise. Responses from those who indicated a lack of relevant expertise were removed from the denominator when evaluating consensus on specific questions. Statements that reached 75% agreement were deemed to achieve consensus. The results from the second round relating to definition of terms are reported elsewhere [5].

Two survey waves were conducted using an online questionnaire administered by the independent company Adelphi Targis.

## Results

Forty-two experts from 18 countries completed the first and 41 completed the second round of the Delphi process. Panel demographics are shown in Table 1.

**TABLE 1:** Expert panel demographics.

Panelist characteristics	First-wave	Second-wave
​	n = 42	n = 41
Age		
18–30 years	0%	0%
31–40 years	9.5%	12.5%
41–50 years	26.2%	22.5%
51–60 years	38.1%	40%
60+ years	26.2%	25%
Gender		
Male	61.9%	65%
Female	38.1%	35%
Country of employment		
Australia	14.3%	15%
Brazil	2.4%	2.5%
Canada	2.4%	2.5%
China	2.4%	2.5%
Finland	2.4%	2.5%
France	14.3%	15%
India	2.4%	2.5%
Ireland	2.4%	2.5%
Italy	7.1%	7.5%
Netherlands	2.4%	2.5%
Portugal	2.4%	2.5%
Slovenia	4.8%	5%
South Africa	4.8%	5%
Spain	14.3%	12.5%
Switzerland	2.4%	2.5%
United Arab Emirates	2.4%	0%
United Kingdom	2.4%	2.5%
United States	14.3%	15%
Primary speciality		
Intensivist/critical care physician	48.3%	50%
Transplant/donation coordinator	15.5%	12.5%
Ethicist or legal scholar	13.8%	14.3%
Anaesthesiologist	8.6%	8.9%
Transplant physician	1.7%	1.8%
Others*	12.1%	12.5%
Transplant surgeon	0%	0%
Primary patient population		
Adult medicine	66.7%	65%
Paediatrics	9.5%	10%
Both adult and paediatric medicine	14.3%	15%
Not applicable to my role	9.5%	10%
Type of hospital in which you work		
Academic hospital	69%	78.4%
Non-academic hospital	7.1%	8.1%
Other academic centres and institutions	23.8%	13.5%
Duration of experience with deceased donation or transplantation		
<5 years	7.1%	7.5%
5–9 years	19%	20%
≥10 years	73.8%	72.5%
Classification of clinical expertise in death diagnosis		
Clinical expertise and experience in diagnosing death in adults	73.8%	72.5%
Clinical expertise and experience in diagnosing death in children	9.5%	10%
I do not have clinical expertise and experience in the diagnosis of death	16.7%	17.5%
Author contributions to academic publications in deceased organ donation or transplantation		
Yes	83.3%	82.9%
No	16.7%	17%
Not applicable to my role	0%	0%
Participation as a principal investigator in a clinical trial relating to deceased organ donation or transplantation		
Yes	33.3%	35%
No	54.8%	52.5%
Not applicable to my role	11.9%	12.5%

Consensus was achieved on 104 statements in the first round, and on a further 11 statements in the second round. The 20 recommendations summarized in Tables 2–4 below reflect the statements for which consensus was achieved, noting some were combined for efficiency.

**TABLE 2:** Consensus recommendations regarding the definition of death.

1. It is important that a single unified definition of death is established worldwide and within individual countries. (See Figure 1)	
2. Death should be defined without regard for deceased donation. Clinical and legal standards for the determination of death should be the same regardless of whether deceased donation will follow a declaration of death	
3. Death should be defined as the permanent absence of brain function	
4. The unifying brain-based concept of death holds that death is ultimately determined by the permanent absence of brain function, which may result from the permanent cessation of brain perfusion, a devastating brain injury, or both	
This provides a connection between circulatory and neurologic determinations of death resulting in a single conceptual pathway for the determination of death	

**TABLE 3:** Consensus recommendations for clinical standards in the determination of death following planned WLSM.

1. The determination of death refers to the process of clinical evaluation used to conclude if the individual is dead or alive
a. Criteria applied in the determination of death refer to the standardized clinical indices used by physicians when diagnosing death b. In the context of standards for the determination of death, the permanent absence of any physiological function (e.g., circulation, brain functions) refers to the absence of function that will not resume spontaneously and will not be restored through intervention
2. Global standardization of clinical criteria for the determination of death, inclusive of prospective adult and pediatric donors in the context of cDCDD, is desirable
a. Clinical standards for determination of death using circulatory criteria following WLSM should be the same for all patients, irrespective of whether cDCDD is planned b. Death determination using circulatory criteria in the context of cDCDD should be in accordance with defined international medical standards, provided these standards are consistent with relevant laws in the jurisdiction c. In the context of cDCDD, where local laws stipulate conditions for the determination of death by circulatory criteria that differ from international medical standards for death determination, legislative efforts to align local law with international standards should be pursued
3. In the context of cDCDD, the determination of death following WLSM should be undertaken by the patient’s treating clinical team or, where relevant, other persons designated by law, not personnel overseeing or involved in the donation process
a. If all physicians involved have relevant expertise and training, the determination of death using circulatory criteria in the context of cDCDD can be made with confidence and integrity, based on a standardised clinical examination(s), at a minimum, by a single physician, who is not involved in the organ recovery, allocation, or transplantation processes for this patient
b. Any physician who is competent to determine death by circulatory criteria following WLSM should be considered competent to do so in the context of cDCDD.
c. Where the protocol for determination of death using circulatory criteria in the context of cDCDD requires use of ancillary tests or other measures in addition to routine standards for determination of death following WLSM, physicians with responsibility for determining death must be trained and assessed as competent in implementing this protocol
4. When death is determined following the planned WLSM, it is important that a minimum time interval should be observed after confirming that circulation is absent, before declaring death (the “no touch period”). This observation period should be sufficient to exclude the possibility that circulation will be restored without medical intervention (autoresuscitation)
a. The mandated minimum period of time that should be observed following circulatory arrest before death is declared and organs can be removed in the context of adult or pediatric cDCDD (the “no touch period”) should be consistent in all countries with cDCDD activity
5. Following WLSM in adults, if circulation has been absent for at least five minutes, spontaneous autoresuscitation will not occur, resulting in permanent absence of perfusion to the brain
a. In a potential cDCDD adult donor, a “no-touch” period of five minutes is sufficient to exclude the possibility of autoresuscitation, thus, in the absence of any intervention that may restart circulation or restore brain perfusion, death can be established. Therefore, five minutes should be the minimum length of the no touch period
6. When determining death by circulatory criteria in the context of adult cDCDD
a. The diagnosis of death requires clinical confirmation of continuous absence of circulation for ≥ five minutes i. An arterial catheter is recommended for detecting the absence of circulation ii. If an arterial catheter is not in place, cannot be placed, or is suspected to be malfunctioning, suitable alternative methods of confirming the absence of circulation include the absence of aortic valve opening by echocardiogram performed by an experienced clinician
b. After five min observation of continuous absence of systemic circulation, a clinical examination to confirm death is needed i. Compared with determination of death following WLSM in the absence of donation, when death is determined in the setting of cDCDD, additional clinical measures to confirm the absence of circulation may be needed
7. In the context of adult cDCDD
a. Cardiorespiratory monitoring should continue following WLSM to enable accurate identification of all important time points, including the onset of circulatory arrest and warm ischemia time b. use of an arterial catheter is recommended to monitor blood pressure and assess FWIT following WLSM. c. Insertion of an arterial catheter is recommended (if not already present) prior to WLSM for detection of loss of pulsatility for determination of death i. Insertion of a radial arterial catheter is recommended where NRP is used post-mortem, to assess absence of reperfusion to arm vessels
8. When the absence of cerebral blood flow is clinically confirmed in an adult patient, this signifies no cerebral perfusion, provided that measures have been taken to prevent reperfusion of the brain in circumstances where NRP is used
a. Clinical confirmation of the permanent absence of cerebral blood flow is sufficient to determine that death has occurred; it is not necessary to test for possible residual neuronal electrical activity (e.g., using EEG)
9. When determining death using circulatory criteria in an adult in the context of cDCDD, clinical best practice should require fulfilment of the apnea criterion assessing neurologic function(s)
a. Absence of cerebral blood flow on cerebral angiogram, transcranial Doppler or nuclear medicine scan is not required

**TABLE 4:** Consensus recommendations regarding the “dead donor rule” and perimortem interventions in the context of cDCDD.

1. The dead donor rule (DDR) refers to the ethical principle, norm, or standard, which holds that
a. Organ donation should not proximately or foreseeably cause the death of the donor i. Interventions which are expected to cause death should not be performed for the purpose of facilitating successful recovery of organs for donation b. Vital organs should only be removed for use in transplantation after the death of the donor c. Death must be lawfully and clinically determined before commencement of removal of donated vital organs
2. Regardless of its legal status, adherence to the DDR is a key principle that healthcare practitioners should always abide by. It remains a central ethical tenet in organ donation
a. Confidence in the integrity of processes for the determination of death in potential organ donors is important to support public trust that the DDR will be respected b. Public trust that the DDR is always respected is essential for the success of deceased donation programs in general and cDCDD programs in particular
3. The DDR is respected (upheld) when removal of organs for donation begins after death has occurred following lawful WLSM or lawful administration of euthanasia (medically assisted death)
4. Surgical removal of organs should never commence until after a person has been lawfully and clinically determined to be dead. A. Although some people may wish for organ recovery to commence before death is declared, in cases where death is inevitable and donation is authorized, this would be a breach of the DDR and should never be permitted.
5. Interventions that are expected to cause death should not be performed for the purpose of facilitating successful recovery of organs for donation
a. Although some people may wish for interventions to be performed prior to death that could hasten death for the purpose of facilitating organ recovery, in cases where death is inevitable and donation is authorized, this would be a breach of the DDR and should never be permitted
6. Interventions that are clinically indicated for the purpose of relieving suffering or improving the quality of end-of-life care should be provided for patients regardless of whether cDCDD is planned
a. When cDCDD is planned, such interventions should be provided for patients, even if the intervention is also expected to facilitate successful recovery of organs for donation
7. Following the lawful determination of death, interventions may be initiated for the purpose of facilitating organ donation without breaching the DDR, provided that measures are taken to prevent restoration of cerebral perfusion
a. Interventions that have the potential to restore cerebral perfusion should not be initiated
b. To ensure that NRP does not breach the DDR, when death is defined by the permanent or irreversible absence of brain function, at a minimum, appropriate techniques must be used to effectively block or divert blood flow to the brain during NRP such that any residual blood flow to the brain is insufficient to restore cerebral perfusion and function

## Discussion

Ethical and clinical uncertainty and the resulting continued controversies regarding the determination of death in the context of cDCDD may profoundly impact donation activities and the associated organ transplant outcomes [9, 10]. In many countries, even in the absence of organ donation, the decision to withdraw life-sustaining measures (WLSM) remains ethically controversial and legally complicated [11–13]. Introducing donation pathways in such environments may exacerbate concerns about conflicts of interest, leading to restrictive measures aimed at preserving trust in the integrity of end-of-life decisions and the diagnosis of death, potentially at the expense of implementing viable cDCDD practices. Fundamentally, the public’s trust is anchored through a definition of death that is conceptually independent of organ donation and transparent in clinical application.

Within this context, it was unsurprising that the results of this study confirm both the importance of establishing consensus on standards for the determination of death in the setting of deceased organ donation and the difficulties of achieving such consensus at the practical level. This is particularly the case in the context of cDCDD, given the wide range of existing laws and policies currently in effect in different countries [14].

**FIGURE 1:**
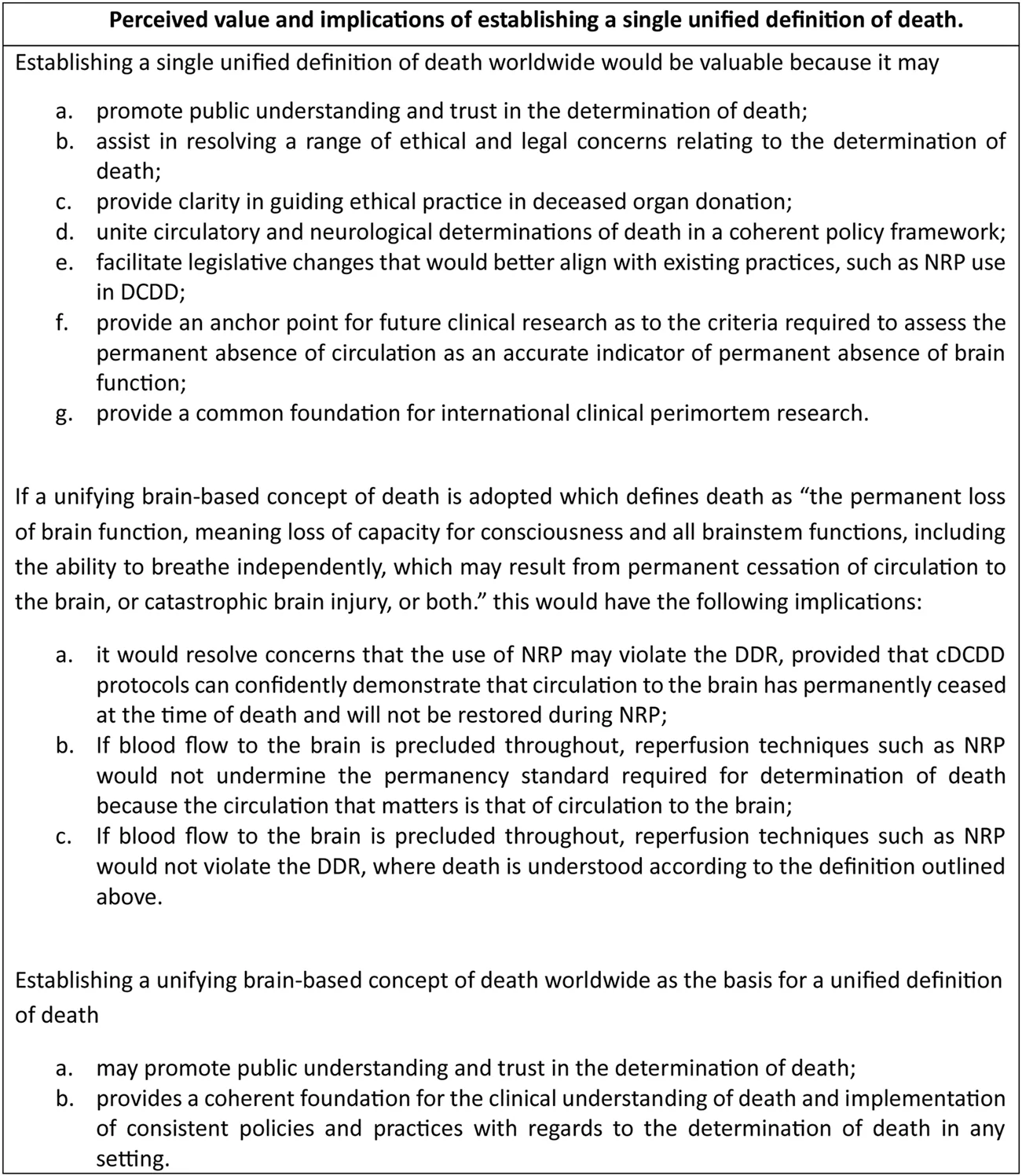
Perceived value and implications of establishing a single unified definition of death.

As shown in Table 2; Figure 1, panellists agreed that establishing a “single unified definition of death worldwide”, notably the “unifying brain-based concept of death”, would be beneficial for several key reasons. However, while there was consensus on the proposed definition of this concept along with some important principles relating to the determination of death and its implications, efforts to achieve consensus on several of the more practical and specific aspects of death determination were less successful. These results nevertheless establish and reaffirm consensus on a number of important principles, including continued support for the DDR, that provide a foundation for current policy and practice. These can be harnessed to guide further efforts to establish minimum clinical standards for the determination of death in the context of cDCDD.

### The definition of death and the importance of universal standards

#### A unifying brain-based concept of death

This account of the unifying brain-based concept of death builds on the work of the 2023 Santander Summit [15], as well as consensus from earlier fora that advocated an “operational” definition of death as “the permanent loss of capacity for consciousness and all brainstem functions … [which] may result from permanent cessation of circulation or catastrophic brain injury” [16, 17]. The notion that death is ultimately determined by the absence of brain functions emerged decades ago [18], but a bifurcated legal standard for defining death by either neurologic or circulatory criteria has persisted in many jurisdictions [15]. This has created uncertainty and inconsistencies between legal standards and clinical practice, in particular when perfusion techniques are implemented that artificially restore circulation to parts of the donor’s body after death has been declared based on a legal definition of death as the permanent cessation of circulation [6, 19]. The unifying concept allows resolution of these inconsistencies by aligning clinical protocols for the determination of death with a universal standard for defining death - *determining* death by the permanent cessation of brain functions or of circulation to the brain and *defining* death as the permanent absence of brain function.

Establishing international consensus on the idea that “death should be defined as the permanent absence of brain function”, independently of subsequent donation, provides a foundational principle for further work that could establish common, standardized brain-based standards for the clinical determination of death. The unifying concept of brain-based death is of particular interest in the context of organ donation, given its implications for *peri mortem* interventions for deceased donation of organs (see below). It may also help to address longstanding controversies arising from distinctions between death determined by neurological or circulatory criteria and thus to promote public trust in the declaration of death [15]. Similarly, consensus on the permanency standard when determining death, consistent with clinical practice in many countries, establishes an impetus for revision of laws and policies that still refer to “irreversibility” [20]. The permanent loss of brain function in determining death is better aligned with current clinical capabilities and cDCDD practices.

The panel, however, did not establish consensus on the recommended definition of death that should be implemented worldwide, but rather on the definition of the unifying brain-based concept of death and the potential value of its adoption. Further work by scientific and legal societies will be needed to explore the implications of adopting this concept as the ultimate legal definition of death and how to parse that from the clinical standards for determining death.

#### Clinical standards for the determination of death

In contrast to the consensus reached on principles relating to death determination, only limited agreement was reached with regards to recommended clinical standards for the determination of death in the context of cDCDD. Despite substantive revisions to the wording of proposed statements and an emphasis placed on minimum rather than ideal standards in the second wave survey, majority agreement was not achieved with regards to the clinical requirements for death determination. Several factors may have influenced these results, such as the diversity of professional expertise, linguistic background, and health system experiences of the panellist population. These include potential bias on the part of panellists with longstanding experience of particular practices or legal requirements in their own jurisdictional context and different interpretations of the framing of statements. Understandably, the clinical expertise, confidence, and familiarity of some panellists with specific end-of-life care practices (e.g., use of continuous deep sedation when WLSM occurs), donation interventions (e.g., use of normothermic regional perfusion to perfuse organs inside the donor’s body after death, prior to surgical recovery [NRP]), and availability of specific technologies or resources (e.g., echocardiography) may have influenced their views on what is feasible or necessary when articulating clinical standards. Dedicated studies aimed at unifying clinical standards for death determination even within countries have notably encountered difficulties, due to the evolution of clinical practices, scientific knowledge, and ongoing debates at the expert level [21].

Consensus was nevertheless established (see Table 3) on several key principles, in particular on two important points with practical implications: the minimum duration of continuous absence of circulation prior to death declaration – the “no-touch” period – being five minutes, and the recommended use of an arterial catheter to verify the cessation of circulation after WLSM in adults in the setting of cDCDD. These recommendations align with the evidence from several observational studies [22], including a large, multicenter study with continuous vital sign monitoring in patients who died after the WLSM, showing that the longest time to any autoresuscitation event was 4 minutes and 20 s [23]. A range of no-touch periods are evident in current cDCDD protocols [24], including from five to 30 min in Europe. [14] This expert consensus should help to reinforce confidence that implementation of a five minute standard prior to declaration of death is appropriate, which could expand opportunities for successful organ recovery and transplantation following cDCDD. It is important to acknowledge with transparency that the five minute standard is based on the best currently available evidence.

The recommended use of continuous invasive arterial blood pressure monitoring for the determination of death and evaluation of functional warm ischaemia time (FWIT) was also important, though the panel did not address a specific threshold of systolic wave amplitude as indicative of cessation of circulation. There was notably no consensus that electrical asystole on electrocardiogram (ECG) monitoring was needed to confirm the cessation of circulation. Further consensus work may help to establish if this reflects agreement that ECG monitoring is redundant when confirming cessation of circulation in the context of cDCDD. This is important because cardiac electrical activity can sometimes continue for a period after permanent circulatory arrest. The mandated use of ECG monitoring for the determination of death in some jurisdictions may thus unnecessarily prolong the time to determine death and increase ischaemic injury to donated organs [14]. The use of ECG monitoring in cDCDD should thus be considered exceptional, for example, when invasive arterial blood pressure monitoring is not possible for technical reasons or is not authorized [25, 26]. There was consensus on the efficacy of noninvasive monitoring devices, and specifically echocardiography, if used by an experienced clinician to detect the absence of aortic valve opening. Conversely, there was consensus that auscultation or palpation should never be used as the sole exam to reliably assess mechanical asystole and lack of circulation for death determination following WLSM in the context of cDCDD.

#### The necessity of additional examinations when determining death in the setting of cDCDD

In addition to well-established practice norms, it is possible that some of the results reflect differences between views on what are necessary requirements or a concern for practical safeguards. For example, there was consensus that a 5-min observation (“no touch”) period of circulatory arrest followed by death declaration was sufficient to preclude autoresuscitation and that “clinical standards for the determination of death should be the same regardless of whether deceased donation will follow a declaration of death.” However, only a minority agreed that no further clinical examination was required at the end of the 5-min period of continuous absence of circulation to confirm death in the setting of cDCDD. This seemingly inconsistent result may stem from lack of clarity over the role of the five minutes of continuous circulatory arrest in confirming permanence - a key principle that is not entirely consistent with existing definitions currently used in many jurisdictions.

Possible reasons for requiring a clinical examination after 5 min of continuous absence of circulation may be to meet regulatory or policy requirements in order to ensure an error in determining death has not been made in cases where organ recovery will commence immediately afterwards. Such an examination may be unnecessary if there has been continuous absence of circulation for 5 min and an adequate method has been used to monitor this, such that any potential resumption of circulation can reliably be excluded. There is physiological evidence that brain function is lost within 30 s of circulatory arrest, or earlier if circulatory arrest follows hypoxic states such as following WLSM, with the brain remaining in a state of permanent non-function if brain perfusion does not resume [27]. Therefore, the inclusion of a circulatory or neurological examination at the end of the period of observation of permanent circulatory arrest would be redundant. There was notably no consensus achieved on any of the options presented that specified potential minimum components of an additional clinical examination to confirm death. Finally, no consensus was obtained, and no clear suggestions emerged about methodological differences, if any, for death determination by circulatory criteria with and without palliative deep sedation during the agonal period before circulatory arrest.

### Ethical standards relating to the determination of death in cDCDD

The results affirm clear support for the DDR and the separation of professional roles when determining death in the context of organ donation as the ethical cornerstones of deceased donation policy and practice despite these standards being debated in philosophical forums. Agreement on the principle that donation should not cause the death of the donor, nor should organ recovery commence prior to the death of the donor (see Table 4) is especially significant at a time when public trust in deceased donation has been shaken by media reports of errors and system failures in donation and transplantation, widespread misinformation about organ donation, general distrust in science, and clear academic disagreement about whether new technologies or interventions such as NRP are inconsistent with the determination of death in the setting of cDCDD [28–30]. Consensus at the level of principles provides an invaluable starting point for those seeking to determine whether particular practices and protocols are ethically acceptable and should motivate further efforts to resolve conflicting directions in practice through scientific investigation and the development of clinical standards [7, 8, 26].

There was consensus that *ante mortem* interventions for the purpose of donation that are expected to cause death are impermissible, even with consent, in order to uphold the DDR; similarly *post mortem* interventions that restore brain perfusion, thus invalidating the diagnosis of death on the basis of permanent absence of brain perfusion, were deemed ethically impermissible. Consistent with the findings of the adult and pediatric pathway groups [7, 8, 31], there was agreement that routine palliative treatments at the end-of-life should be provided as needed, irrespective of whether cDCDD is intended. This provides valuable reassurance to clinicians that provision of necessary palliative care, which in some cases may have secondary effects that influence the outcomes of cDCDD, should not be avoided for fear of breaching the DDR. Rather than dictating specific *ante* or *postmortem* interventions or practices as ethically permissible or impermissible, the standards articulated herein should guide decision-making when evaluating interventions in light of the relevant evidence and in specific clinical contexts. In particular, the results support the use of *post mortem* NRP, provided that measures are taken to prevent restoration of cerebral perfusion consistent with the unified concept of death. The adequacy of specific measures and clinical tools used in evaluating their efficacy has yet to be determined [32].

## Conclusion

In summary, the consensus results provide a starting point for aligning clinical practices worldwide around the definition of death based on the foundational unified concept of death and a clinical standard of permanence. Further, consensus affirms that two key requirements readily achievable in the hospital environment can establish the permanent cessation of brain function that is needed to determine death after WLSM, including in potential adult cDCDD donors: 1) the intra-arterial pressure monitoring or the echocardiogram show that the systemic circulation is absent; 2) the duration of continuous absence of circulation of five minutes is sufficient for death determination if no action will be performed that will restore brain perfusion. If these requirements are fulfilled, the patient’s clinical status satisfies the definition of death, is dead and may be properly declared dead, regardless of any procedures or actions that may take place in the deceased body, thereafter, provided these do not restore cerebral perfusion.

With consensus on these foundational points and acknowledgement of the value and need for further standardization in definitions and clinical protocols, work should continue to drive future consensus. This future work should strive to unify the circulatory and neurological determinations of death in a coherent policy framework by ensuring standardized clinical practice is consistent with the unified concept of death. Ultimately, once greater consensus is achieved, this will facilitate legislative changes in each country that will enable better alignment and support of existing practices, such as NRP, as well as future innovations in deceased donation. While each of these steps towards consensus are time intensive, the process will ultimately help advance and harmonize legislations and regulations on death determination, thereby fostering public and professional trust in organ donation and transplantation worldwide.

## Data Availability

The raw data supporting the conclusions of this article will be made available by the authors, without undue reservation.

## References

[1] Murphy NB Shemie SD Capron A Truog RD Nakagawa T Healey A Advancing the scientific basis for determining death in controlled organ donation after circulatory determination of death. Transplantation (2024) 108:2197–208. 10.1097/tp.0000000000005002 38637919 PMC11495540

[2] Murphy NB Slessarev M Basmaji J Blackstock L Blaszak M Brahmania M Ethical issues in normothermic regional perfusion in controlled organ donation after determination of death by circulatory criteria: a scoping review. Transplantation (2024) 109:597–609. 10.1097/TP.0000000000005161 39192464 PMC11927451

[3] Nielsen BEJ Mjaaland MT . Does controlled donation after circulatory death violate the dead donor rule? Am J Bioeth (2023) 23(2):4–11. 10.1080/15265161.2022.2040646 35238715

[4] Dominguez-Gil B López-Fraga M Muller E Potena L Martin DE Pérez Blanco A Santander global summit in transplantation: supporting global convergence in the shared goals of sufficiency, transparency and oversight. Transplantation (2024) 109:2–6. 10.1097/TP.0000000000005222 39437365

[5] Martin D Cillo U Berman M Glazier AK Miñambres E Opdam H Seeking global agreement on controlled donation after circulatory determination of death: methodology and definitions of the bucharest international European society for organ transplantation (ESOT) consensus. Transpl. Int. (2026) 39:16280. 10.3389/ti.2026.16280 42294092 PMC13254459

[6] Bernat JL Domínguez-gil B Glazier AK Gardiner D Manara AR Shemie S Understanding the brain-based determination of death when organ recovery is performed with DCDD *in situ* normothermic regional perfusion. Transplantation (2023) 107(08):1650–4. 10.1097/TP.0000000000004642 37170405

[7] Opdam H Pérez-A Gardiner D Hasz R Jansen NE Le Dorze M Establishing standards for controlled donation after circulatory determination of death in adults: the Bucharest international European society for organ transplantation consensus. Transpl. Int. (2026) 39:16284. 10.3389/ti.2026.16284 42326389 PMC13276612

[8] Weiss MJ Siebelink MJ Cavazzoni E Figini MA Kazzaz Y Nadalin S The Bucharest international European society for organ transplantation consensus on paediatric controlled donation after circulatory determination of death. Transpl. Int. (2026). 39:16462. 10.3389/ti.2026.16462 42453307 PMC13367135

[9] O’Rourke J Crowe G Turner R Gaffney A . Donation after circulatory death: a narrative review of current controversies, attitudes, and the evolving role of regional perfusion technology. AME Med J (2024) 9:9. 10.21037/amj-23-65

[10] da Graca B Borries T Polk H Ramakrishnan S Testa G Wall A . Ethical issues in donation following circulatory death: a scoping review examining changes over time from 1993 to 2022. AJOB Empir Bioeth (2023) 14(4):237–77. 10.1080/23294515.2023.2224590 37343208

[11] Pérez Castro P Salas SP . Ethical issues of organ donation after circulatory death: considerations for a successful implementation in Chile. Dev World Bioeth (2022) 22(4):259–66. 10.1111/dewb.12338 34773430 PMC9886168

[12] Mueller PS . Ethical and legal concerns associated with withdrawing mechanical circulatory support: a U.S. perspective. Front Cardiovasc Med Front Media S.A. (2022) 9:897955. 10.3389/fcvm.2022.897955 35958394 PMC9360408

[13] Avidan A Sprung CL Schefold JC Ricou B Hartog CS Nates JL Variations in end-of-life practices in intensive care units worldwide (Ethicus-2): a prospective observational study. Lancet Respir Med (2021) 9(10):1101–10. 10.1016/S2213-2600(21)00261-7 34364537

[14] Lomero M Gardiner D Coll E Haase-Kromwijk B Procaccio F Immer F Donation after circulatory death today: an updated overview of the European landscape. Transpl Int (2020) 33(1):76–88. 10.1111/tri.13506 31482628

[15] Gardiner D McGee A Kareem Al Obaidli AA Cooper M Lentine KL Miñambres E Developing and expanding deceased organ donation to its maximum therapeutic potential: an actionable global challenge from the 2023 Santander summit. Transplantation (2025) 109(1):10–21. 10.1097/TP.0000000000005234 39437375

[16] Shemie SD Hornby L Baker A Teitelbaum J Torrance S Young K International guideline development for the determination of death. Intensive Care Med (2014) 40(6):788–97. 10.1007/s00134-014-3242-7 24664151 PMC4028548

[17] Gardiner D Shemie S Manara A Opdam H . International perspective on the diagnosis of death. Br J Anaesth (2012) 108(1):i14–i28. 10.1093/bja/aer397 22194427

[18] Diagnosis of death. Memorandum issued by the honorary secretary of the conference of medical royal colleges and their faculties in the United Kingdom on 15 January 1979. BMJ: Br Med J (1979) 1:332. 10.1136/bmj.1.6159.332 421104 PMC1597667

[19] Glazier AK Capron AM . Normothermic regional perfusion and US legal standards for determining death are not aligned. Am J Transplant (2022) 22(5):1289–90. 10.1111/ajt.17002 35170218

[20] Bernat JL Capron AM Bleck TP Blosser S Bratton SL Childress JF The circulatory-respiratory determination of death in organ donation. Crit Care Med (2010) 38(3):963–70. 10.1097/CCM.0b013e3181c58916 20124892

[21] Murphy NB Hartwick M Wilson LC Simpson C Shemie SD Torrance S Rationale for revisions to the definition of death and criteria for its determination in Canada. Can J Anesth (2023) 70(4):558–69. 10.1007/s12630-023-02407-4 37131021 PMC10203013

[22] Zorko DJ Shemie J Hornby L Singh G Matheson S Sandarage R Autoresuscitation after circulatory arrest: an updated systematic review. Can J Anesth/j Can Anesth (2023) 70:699–712. 37131027 10.1007/s12630-023-02411-8 PMC10202982

[23] Dhanani S Hornby L van Beinum A Scales NB Hogue M Baker A Resumption of cardiac activity after withdrawal of life-sustaining measures. New Engl J Med (2021) 384(4):345–52. 10.1056/nejmoa2022713 33503343

[24] Dhanani S Hornby L Ward R Shemie S . Variability in the determination of death after cardiac arrest: a review of guidelines and statements. J Intensive Care Med (2012) 27(4):238–52. 10.1177/0885066610396993 21841147

[25] Klowak JA Nguyen AL Malik A Hornby L Doig CJ Kawchuk J Diagnostic test accuracy for cessation of circulation during death determination: a systematic review. Can J Anesth (2023) 70:671–84. 37138156 10.1007/s12630-023-02424-3 PMC10202983

[26] Shemie SD Wilson LC Hornby L Basmaji J Baker AJ Bensimon CM A brain-based definition of death and criteria for its determination after arrest of circulation or neurologic function in Canada: a 2023 clinical practice guideline. Can J Anesth/J Can d’anesthésie (2023) 70(4):483–557. 37131020 10.1007/s12630-023-02431-4 PMC10203028

[27] Shemie SD Gardiner D . Circulatory arrest, brain arrest and death determination. Front Cardiovasc Med (2018) 5:15. 10.3389/fcvm.2018.00015 29662882 PMC5890102

[28] Rosenthal BM Tate J . A push for more organ transplants is putting donors at risk. New York Times (2025). Available online at: https://www.nytimes.com/2025/07/20/us/organ-transplants-donors-alive.html (Accessed February 18, 2026).

[29] Esbensen KL DeCamp M Crigger EJ Snyder Sulmasy L Esbensen KL ACP Ethics, Professionalism and Human Rights Committee. Ethical issues in organ transplantation: a position paper from the American college of physicians. Ann Intern Med (2025) 178(12):1772–8. 10.7326/ANNALS-25-01738 41144970

[30] Peled H Mathews S Rhodes D Bernat JL . Normothermic regional perfusion requires careful ethical analysis before adoption into donation after circulatory determination of death. Crit Care Med (2022) 50(11):1644–8. 10.1097/CCM.0000000000005632 36227032

[31] Miñambres E Berman M Antonini MV Campo-Cañaveral De La Cruz JL Croome K Feltrin G Normothermic regional perfusion (NRP) use in controlled donation after circulatory determination of death (cDCDD): results of the European Society for Organ Transplantation Bucharest consensus conference. Transpl. Int. (2026). 39:16391. 10.3389/ti.2026.16391 42558417 PMC13437514

[32] Shemie SD Watson CJ . Normothermic regional perfusion in donation after circulatory determination of death—Confirming the absence of brain reperfusion. Am J Transplant (2025) 25(8):1596–8. 10.1016/j.ajt.2025.05.031 40441301

